# The Role of RUNX2 in Osteosarcoma Oncogenesis

**DOI:** 10.1155/2011/282745

**Published:** 2010-12-09

**Authors:** J. W. Martin, M. Zielenska, G. S. Stein, A. J. van Wijnen, J. A. Squire

**Affiliations:** ^1^Department of Pathology and Molecular Medicine, Queen's University, Kingston, ON, Canada K7L 3N6; ^2^Department of Pathology and Laboratory Medicine, Hospital for Sick Children, Toronto, ON, Canada M5G 1X8; ^3^Department of Cell Biology, University of Massachusetts Medical School, Worcester, MA 01655, USA

## Abstract

Osteosarcoma is an aggressive but ill-understood cancer of bone that predominantly affects adolescents. Its rarity and biological heterogeneity have limited studies of its molecular basis. In recent years, an important role has emerged for the RUNX2 “platform protein” in osteosarcoma oncogenesis. RUNX proteins are DNA-binding transcription factors that regulate the expression of multiple genes involved in cellular differentiation and cell-cycle progression. RUNX2 is genetically essential for developing bone and osteoblast maturation. Studies of osteosarcoma tumours have revealed that the RUNX2 DNA copy number together with RNA and protein levels are highly elevated in osteosarcoma tumors. The protein is also important for metastatic bone disease of prostate and breast cancers, while RUNX2 may have both tumor suppressive and oncogenic roles in bone morphogenesis. This paper provides a synopsis of the current understanding of the functions of RUNX2 and its potential role in osteosarcoma and suggests directions for future study.

## 1. Introduction

Osteosarcoma is an aggressive cancer of bone with unknown etiology and often poor clinical outcome. It is the most common primary malignant tumour of bone, representing about 35% of bone cancer cases [1], and it predominantly affects individuals in their second decade of life. Most often, tumours arise from osteoid-producing neoplastic cells in the metaphyses of the long bones, including the distal femur and proximal humerus [1], and less commonly in the axial skeleton and other nonlong bones [2]. Tumours frequently possess cells with extensive, complex genomic rearrangements, and few consistent changes have been observed across this heterogeneous disease. 

No molecules for targeted therapy have been developed for osteosarcoma, and survival rates have not improved for several decades since the introduction of chemotherapy to treatment of the disease (reviewed in [3]). The current standard of care comprises limb-sparing surgery and combination neoadjuvant chemotherapy consisting of high dose methotrexate, doxorubicin, cisplatin, and ifosfamide [4]. Treatment of the bone tumours prior to the use of chemotherapy was solely surgical with a higher percentage of cases undergoing amputation and with an associated 5-year survival of about 15% [3, 5].

Ongoing studies continue to detect genes whose protein products may play a role in osteosarcoma oncogenesis and may have potential as therapeutic targets. The tumour suppressors p53 and pRB are inactivated at the DNA level in roughly 50%–70% of sporadic osteosarcomas [6], and germline inactivations of either of those proteins significantly increase risk for developing osteosarcoma [6, 7]. For example, Li-Fraumeni patients, who have p53 germ line mutations, have an increased incidence of osteosarcoma [8, 9]. A similar situation arises with RecQL helicase inactivations [6], which are also associated with chromosomal instability in osteosarcoma tumours [10]. This tumour is also characterised by a vastly heterogeneous array of complex genomic rearrangements, but their description is beyond the scope of this paper and can be retrieved in reports by our lab and others [11–21]. 

For the purpose of this paper, it will suffice to call attention to the chromosomal region 6p12-p21, which encompasses the *RUNX2 *gene and experiences recurrent gain and amplification in osteosarcoma [11–17, 22]. In our lab, we have detected amplification-related overexpression of the *RUNX2* gene in a subset of osteosarcoma tumours and identified a correlation between high *RUNX2 *mRNA overexpression and poor tumour response to chemotherapy based on the percentage of tumour necrosis following treatment [23]. This prospective estimate of response is an indirect predictor of response that is routinely used as part of patient management. In a separate retrospective cohort of osteosarcoma patient specimens, we have also detected correlations between copy number gain of *RUNX2 *and poor tumour necrosis in response to chemotherapy (measured by fluorescence *in situ *hybridisation) and between high RUNX2 protein levels and poor chemoresponse in the tumours [paper in preparation]. Furthermore, RUNX2 protein levels appear to be selectively deregulated in several osteosarcoma-derived cell culture models [24–27]. *RUNX2/*RUNX2 thus has potential as a predictive biomarker for osteosarcoma, but a better understanding of the gene and protein in the context of the disease is necessary before considering targeted treatments and diagnostic, prognostic, and predictive tests.

## 2. RUNX Family of Transcription Factor Genes

The three members of the mammalian *RUNX* family of tissue-specific transcription factor genes encode the DNA-binding *α* components of the core-binding factor (CBF) complex [28]. In the literature, the genes are also known by the family names *core-binding factor-*α**(*CBFA*), *acute myeloid leukemia *(*AML*), and mouse *polyoma enhancer-binding protein 2*α** (*PEBP2*α**), depending on the context of their study [29]. The RUNX proteins, as part of the CBF complex, regulate differentiation, survival, and growth in a variety of tissues, but are specifically essential for definitive hematopoiesis (RUNX1), osteogenesis (RUNX2), as well as neurogenesis and gut development (RUNX3) (reviewed in [30]). *RUNX1/AML1/CBFA2/PEBP2*α*B* was discovered as a common chromosomal translocation target in chronic myelogenous and acute myeloid leukemias (reviewed in [31]), and its critical necessity for adult blood-cell production was discovered in *RUNX1*-null mice, which lacked definitive hematopoiesis [32, 33]. *RUNX3/AML2/CBFA3/PEBP2*α*C *expression is necessary for development of neuronal networks [34, 35] and the gastrointestinal tract [36], and its inactivation is strongly associated with gastric cancer [37]. *RUNX2/AML3/CBFA1/PEBP2*α*A* encodes an essential determinant of osteoblast differentiation [38, 39] that regulates the expression of many genes during bone development (reviewed in [40]).

## 3. RUNX2 Structure-Function Relationship

**Figure 1:**
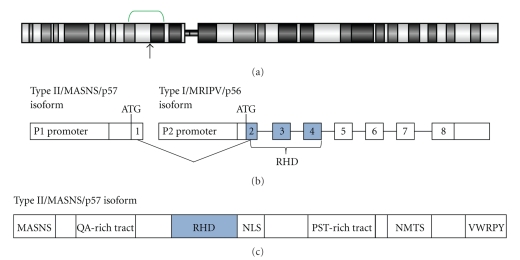
Chromosome 6 and *RUNX2/*RUNX2. (a) Chromosome 6 and location of *RUNX2*. The green bracket approximately spans the minimal common region of gain identified by array comparative genomic hybridisation (aCGH) studies of osteosarcomas, between cytobands 6p21.2 to 6p12.3 (spanning nucleotide positions 36,800,000 bp to 51,100,000 bp, resp.). All genomic information was obtained from UCSC Genome Browser (http://genome.ucsc.edu/), March 2006 (hg18) assembly. (b) Gene structure of *RUNX2*. Major isoforms MASNS and MRIPV are transcribed starting from promoters P1 and P2, respectively, and ATG indicates the start codon. The MRIPV isoform is encoded from exons 2–8, while the MASNS isoform is encoded from all eight exons. The Runt homology domain (RHD) is encoded from portions of exons 2, 3 and 4 (shaded). (c) Protein structure of RUNX2. The Type II/p57 isoform comprises 521 amino acids and begins with the bone-specific N-terminal MASNS polypeptide. It has a glutamine/alanine (QA) rich tract and a proline/serine/threonine (PST) rich tract that are both unique to RUNX2 in the RUNX family of proteins. The protein also possesses the RHD DNA-binding domain, the nuclear-localisation signal (NLS), the nuclear matrix targeting signal (NMTS), and the C-terminal VWRPY domain for TLE/Groucho corepressor interactions. Adapted from [44, 45, 49, 50].

The *RUNX2 *gene occupies approximately 220 kbp on chromosome 6 near the border between cytobands 6p21.1 [28, 41] and 6p12.3 (UCSC Genome Browser, March 2006 hg18 assembly), and the RUNX2 protein exists as two major isoforms [42] (Figure 1). Two distinct promoters for the *RUNX2 *gene, P1 and P2, give rise to two biologically unique transcripts [43] (Figure 1(b)), and alternative splicing contributes to at least three variants of the protein based on the at least eight exons known to make up the gene [41, 44] (Figure 1(b)). The *RUNX2 *gene is a unique member of the *RUNX* family in that it produces the largest protein product (521 amino acids) [45], which possesses two domains distinct from its homologues: a short stretch of glutamine-alanine (QA) repeats at the N-terminus and a C-terminal proline/serine/threonine (PST) rich tract, both regions of which are necessary for full transactivation activity [46]. However, the protein has high-sequence identity with the other RUNX proteins, sharing with them the DNA-binding Runt domain, the nuclear localisation signal (NLS), the nuclear matrix targeting signal (NMTS), and a C-terminal VWRPY sequence, which allows interaction with corepressors transducin-like enhancer of split (TLE)/Groucho [47, 48] (Figure 1(c)). 

The Runt domain is common among the RUNX proteins [51], and was first characterised in the Runt and Lozenge proteins of *Drosophila*, in which they are essential for the regulation of many developmental processes, including segmentation, sex determination, and hematopoiesis (reviewed in [52]). This domain confers the ability for binding to DNA and for heterodimerisation with CBF*β* [53] to form the CBF complex. The CBF*β* protein, though necessary for RUNX activity, does not directly affect transcription regulation itself, but rather allosterically increases the DNA-binding capacity of its RUNX partner [54, 55].

 RUNX2 binds specific *cis*-acting elements via the conserved Runt domain to enhance transcription of genes in many tissues during embryogenesis, particularly in T-lymphocytes throughout development of the thymus [56] and developing cartilage [57]. However, its most significant function is in the regulation of osteoblast differentiation during bone development [45].

## 4. Importance of RUNX2 in Normal Skeletal Development

The significance of RUNX2 in skeletal development was first suggested by studies of the autosomal dominant disease cleidocranial dysplasia (CCD). Initially, linkage studies of kindreds with CCD led to the discovery that a single locus within cytoband 6p21 was associated with the disease [58, 59]. Higher resolution cytogenetic and sequencing analyses subsequently identified several mechanisms for heterozygous inactivation of the *RUNX2 *gene: in-frame polyalanine expansions within the QA domain, heterozygous deletions due to chromosomal inversion, nonsense mutations, missense mutations, and frameshift mutations due to insertion or microdeletion, all of which resulted in *RUNX2 *haploinsufficiency [60, 61]. Mouse studies demonstrated conclusively that *RUNX2 *was necessary for normal bone development. Mice heterozygous for mutant *RUNX2 *recapitulate human CCD, and mice homozygous for mutant *RUNX2 *were deficient in osteoblasts and vascularisation of marrow due to a lack of osteoblast and endothelial differentiation of periosteal mesenchymal stem cells (MSCs) [38, 39, 62, 63].

**Figure 2:**
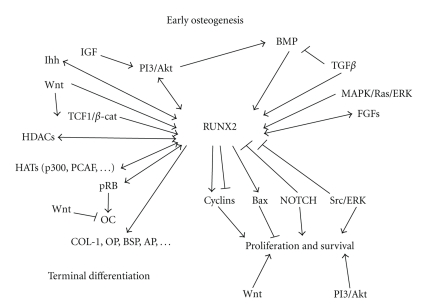
*RUNX2 *transcription and RUNX2 activity are influenced by many signaling molecules during osteoblast development. Summarised here, a large number of complex protein-protein interactions characterise RUNX2 activity, and transcription of *RUNX2* and protein levels of the encoded product are influenced by a multitude of factors depending on the stage of osteoblast differentiation (see text for detailed descriptions). Arrows indicate protein-protein interactions and/or transcriptional upregulation whereas connections ending with a flat arrowhead indicate inhibitory effects.

In its capacity as a transcription factor necessary for osteoblast differentiation [64, 65] and full skeletal development [38, 39], RUNX2 acts as a “platform protein,” in that it interacts with a variety of coactivator and corepressor proteins, including chromatin remodeling factors and epigenetic modifiers (reviewed in [45]). Transcriptional regulation of *RUNX2* is also complex and affected by a variety of signaling pathways (a summary of protein-protein interactions and transcriptional regulators of *RUNX2 *is shown in Figure 2). The complexity of RUNX2 signaling is further compounded by its autorepression [49], by its presence in at least two isoforms, and by its emerging relevance in the development of nonosteogenic cells [66].

## 5. Upstream Signaling and Transcription Regulation of RUNX2

Discrete *RUNX2* transcriptional activity is necessary for all stages of osteogenesis, and expression of the MASNS/p57 (Type II) isoform from the osteoblast-specific P1 promoter leads to the osteoblast-specific isoform of the protein [67]. The MRIPV/p56 (Type I) isoform of RUNX2, expressed from the chondrocyte-specific P2 promoter [68], is required for chondrocyte hypertrophy and maturation, in a role subject to repression by the chondrocyte-specific transcription factor SOX9 [69, 70]. Upstream *RUNX2 *promoter elements bind a variety of factors which form important branches of embryogenic pathways, including Hedgehog (Hh), canonical Wnt, mitogen-activated protein kinase (MAPK), fibroblast growth factor (FGF), and bone morphogenetic protein (BMP)/transforming growth factor *β* (TGF*β*) (Figure 2). 

During endochondral ossification, one of the first events to begin differentiation of osteoprogenitor cells from MSCs is the transcriptional activation of *RUNX2 *by Indian hedgehog (Ihh) [71, 72], which is itself upregulated by RUNX2 [73]. Other essential signals are the insulin-like growth factors (IGFs), which are implicated in early osteogenesis. IGF signaling activates the phosphatidylinositol 3-kinase (PI3K)-Akt pathway, with AKT2 being required for both BMP2 signaling and for *RUNX2 *transcriptional activation [74, 75]. The canonical Wnt protein T-cell factor 1 (TCF1), with betacatenin, also upregulates *RUNX2 *expression in MSCs [76], but further studies have shown that Wnt signaling is most critical in the transition from RUNX2+Osterix1− osteoprogenitors to RUNX2+Osterix1+ cells [77], and in subsequent osteoblast maturation [72]. 

During progression of osteogenesis, numerous other factors regulate the expression of *RUNX2*. SP1, ETS1, and ELK1 all stimulate *RUNX2 *expression, the former two predominating during osteoblast proliferation and early differentiation, and the latter protein maintaining basal *RUNX2* transcriptional activity in later stages of differentiation [78]. Transcriptional activation of *RUNX2 *is also facilitated by the BMP2 signaling cascade via the homeodomain proteins DLX3 and DLX5 [79] and by MAPK/Ras/ERK signaling in response to mechanical stress [80, 81]. FGFs stimulate bone formation through the protein kinase C (PKC) pathway, with FGF2/FGFR2 activating expression of *RUNX2*, as well as transcriptional activity of the RUNX2 protein [82]. 

On the other hand, expression of *RUNX2* is reduced by 1,25-(OH)_2_-vitamin D3 (VD3) [83], peroxisome proliferation-activated receptor gamma 2 (PPAR*γ*2) [84], and tumour necrosis factor alpha (TNF*α*) at the transcriptional and posttranscriptional levels [85]. NKX3.2/BAPX1 is upregulated by SOX9 in terminal chondrogenesis to reduce expression of *RUNX2* [86, 87]. Cyclic AMP signaling promotes proteasome-mediated degradation of RUNX2 [88], and RUNX2 activity is modulated by residue-specific phosphorylation [89], binding by inhibitory proteins such as coactivator activator (CoAA) [90], and acetylation of the protein [91].

## 6. RUNX2 Signaling in Osteogenesis Has Potential for Deregulation in Oncogenesis

RUNX2 regulates osteoblast lineage determination and expansion, osteoblast maturation, and terminal differentiation via a complex variety of pathways. Early osteoblast progenitor cells arise from pluripotent MSCs due to direct interactions of RUNX2 with broadly acting developmental pathways. Canonical Wnt factors and Hh family members are well known to inhibit adipogenic or chondrogenic differentiation of MSCs and to promote a preosteoblastic phenotype [92–94]. A number of relationships between RUNX2 and the canonical Wnt pathway have recently been shown to guide osteoblast commitment. In MSCs, RUNX2 forms a complex with lymphoid enhancer-binding factor 1 (LEF1), which is coactivated by betacatenin, to activate the *fibroblast growth factor 18* (*FGF18*) gene [95], whose product inhibits chondrogenesis and supports osteogenesis [96]. 

The canonical Wnt pathway in particular is important throughout osteoblast differentiation. Without Wnt signaling, RUNX2-mediated transcriptional activation of the *osterix *(*Osx1/SP7*) gene in osteoprogenitors cannot lead to further commitment to the osteoblast lineage [97]. Following lineage commitment, RUNX2 promotes differentiation, and a particularly important early step following commitment is the interaction between RUNX2 and SMAD proteins induced by BMP and TGF*β*. In osteoprogenitors, BMP2 serves to induce *osterix* expression and promote osteoblast differentiation in a RUNX2-independent manner [98, 99], and in order for osteogenesis to approach completion, BMP/TGF*β* signaling must be facilitated by the formation of the RUNX2-SMAD complex, which activates transcription of late osteoblast markers [100].

Proliferation and migration of committed osteoblasts precedes quiescence and terminal differentiation. Osteoblast proliferation and survival is promoted in large part by canonical Wnt signaling directly through LRP5 [101, 102] and indirectly via Src/ERK and PI3K/Akt [103]. Several studies have shown that RUNX2 attenuates osteoblast proliferation, and its protein levels are maximal during the G1 phase in which differentiation and growth occur. RUNX2 activity is maintained at high levels into the G0 phase if quiescence is induced, but is otherwise downregulated at the G1 to S transition and in the subsequent S, G2, and M phases [24, 89, 104]. Mitosis sees residual RUNX2 localised in active nucleolar organising regions to repress transcription of ribosomal RNA genes [105]. RUNX2 may support epigenetic regulation of protein-encoding genes during mitosis [106], a mechanism referred to as “bookmarking” [107]. *In vitro, *contact inhibition or serum deprivation is associated with increased RUNX2 and cell-cycle exit, while RUNX2 deficiency induces increased growth potential [104]. Through activation by BMP/SMAD signaling, RUNX2 upregulates *BAX *expression to induce apoptosis in studies of the osteosarcoma cell line SAOS-2 [108].

Though its role in cell growth inhibition is well established, RUNX2 also promotes cell proliferation and survival. The maximal levels of RUNX2 during G1 may actually be necessary to stimulate continued cell division [24, 109]. RUNX2 represses transcription of *p21/CDKN1A/WAF1/CIP1*, which encodes a cyclin-dependent kinase inhibitor that arrests cells in G1 [110], and it activates *Gpr30 *transcription and represses *Rgs2* transcription to increase cellular response to mitogenic signaling through cyclic AMP and G-protein-coupled receptor signaling pathways [109]. In converse to the finding that RUNX2 upregulates *BAX *expression in the SAOS-2 cell line [108], nitric oxide (NO) treatment of the MG-63 osteosarcoma cell line induces RUNX2-mediated *BCL2 *expression, which promotes survival of the cells during oxidative stress [111]. NO signaling through cyclic guanosine 3′,5′-monophosphate (cGMP) may also cause site-specific phosphorylation of RUNX2 by protein kinase G (PKG), leading to upregulated transcription of the matrix metalloproteinase (MMP) gene *MMP13 *[112]. MMP13 is one of several members of the MMP family with important roles in cartilage degradation during endochondral ossification and later bone remodeling (reviewed in [113]).

Additionally, during bone development and remodeling, RUNX2 and PI3K-Akt mutually upregulate each other to enhance chemotactic osteoblast migration [114], which occurs along gradients of platelet-derived growth factor (PDGF), TGF*β*, and IGF [115–117]. Terminal osteoblast differentiation is accomplished through cell-cycle exit and complete expression of osteoblast phenotypic markers. RUNX2 induces higher levels of p27*^KIP1^*/CDKN1B, which inhibits S-phase cyclin-dependent kinases to promote cell-cycle exit and causes dephosphorylation of pRB [118]. Active, hypophosphorylated pRB is necessary for cell-cycle exit at this stage [119] and, through cooperation with the transcription factor HES1 [120], the hypophosphorylated form of pRB is bound by RUNX2. The RUNX2-pRB complex then coactivates transcription of genes encoding late markers of osteoblast differentiation, including *osteocalcin* [121]. *Osteocalcin *is also activated by RUNX2 in complex with histone acetyltransferases (HATs) p300 and p300/cyclic AMP receptor element-binding protein binding protein-associated factor (PCAF) [122], as well as monocytic leukemia zinc finger protein (MOZ) and MOZ-related factor (MORF) [123]. Other late osteoblast markers include alkaline phosphatase (AP), osteopontin (OP), bone sialoprotein (BSP), and collagen type I (COL-1), all of which require RUNX2-SMAD signaling, induced by BMP/TGF*β*, to be expressed [100] (Figure 2).

Depending on the phosphorylation level of RUNX2 and the stage of differentiation, it also interacts with several corepressor proteins. Histone deacetylases (HDACs) 6 and 3 interact with RUNX2 to repress *p21/CDKN1A/WAF1/CIP1* and *osteocalcin*, thus regulating osteoblast development during proliferation and terminal differentiation [110, 124]. The mSin3a, TLE/Groucho, and Yes-associated protein (YAP) corepressors form complexes with RUNX2 and other HDAC proteins to repress expression of osteoblast-specific genes, particularly *osteocalcin* [47, 125, 126], and HDAC4 induces transcriptional repression by binding RUNX2 to inhibit its intrinsic DNA-binding activity [127]. The transcriptional regulation and tissue-specific nature of RUNX2 activity thus depends a great deal on the proteins it forms multisubunit complexes with, and studies are ongoing to characterise the complex relationship between RUNX2 and the downstream factors that control osteoblast development.

## 7. Potential Significance of RUNX2 in Osteosarcoma

**Figure 3:**
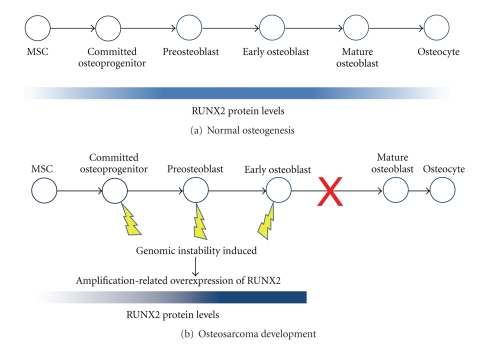
Osteoblast differentiation and RUNX2 protein levels. (a) In normal osteogenesis initiating in MSCs, overall RUNX2 protein levels are maximal in preosteoblasts and early mature osteoblasts, after gradually increasing during commitment. Overall RUNX2 levels are very low in mature osteoblasts and osteocytes [129]. RUNX2 activity and levels are modulated according to cell-cycle stage by posttranslational modification and transcriptional regulation of *RUNX2*, respectively. (b) In osteosarcoma development, genomic instability is induced (lightning bolts), for example by inactivation of pRB or p53, in cells committed to the osteoid lineage. Extensive rearrangements occur, with amplification of chromosome 6p12-p21 being a frequent early event in many cases. Amplification-related overexpression of *RUNX2* could result, leading to high levels of RUNX2 protein throughout the cell cycle and disrupted regulation of RUNX2 activity. Consequently, osteoblast differentiation is halted before or during maturation and characteristics of immature osteoblast-like cells are retained in the resulting osteosarcoma. Adapted from [130].

During development of normal bone, RUNX2 levels increase gradually after commitment of MSCs to the osteoblast lineage to maximal levels in early osteoblasts (Figure 3(a)). Several recent studies of osteosarcoma specimens have reported constitutively high protein levels of RUNX2. Although such studies of RUNX2 in clinical samples are rare, they are compelling in their findings. Andela et al. [128] published the earliest report we could find of RUNX2 immunoreactivity in osteosarcomas; the researchers tested 11 pathology specimens of the cancer and found RUNX2 immunopositivity in all of them. A comprehensive DNA-mRNA-protein analysis of patient samples by Lu et al. [12] found mRNA overexpression of *RUNX2* in 13 of 13 samples with genomic amplification in 8 of the 13. 

Three more recently published studies were successful in linking *RUNX2 *expression with measures of clinical course in patients with osteosarcoma. In a study of 22 osteosarcomas by our lab, mRNA overexpression of *RUNX2 *was on average 3.3 times higher in tumours that had responded poorly (<90% necrosis) to neoadjuvant chemotherapy relative to tumours with good response (>90% necrosis). Compared to normal human osteoblasts, every tumour specimen had higher *RUNX2 *mRNA expression [23]. Similarly, Won and colleagues observed low RUNX2 expression in 60% (29/48) of cores and high RUNX2 expression in 23% (11/48) of cores. In this study, high RUNX2 expression was significantly correlated with metastasis and predicted a trend towards lower survival [131]. Another study analysed the comparative immunoreactivity of RUNX2 in different types of patient samples, finding positive staining in 60% (12/20) of biopsy samples and 73% (8/11) of metastatic tumours. Interestingly, this same study found only 16% (4/25) of postchemotherapeutic resections were positive for RUNX2 staining [132]. Thus, the results of these recent studies are suggestive of predictive value of RUNX2.

The function of RUNX2 in osteosarcoma has not yet been identified, but given the complex functionality of RUNX2 in developing osteoblasts, deregulation of the protein could act during osteosarcoma pathogenesis. Significantly, cell cycle-dependent regulation of RUNX2 is absent in the cell line SAOS-2 and the protein is maintained at high levels throughout the cell cycle, particularly during the G1 to S transition when it is normally downregulated [24]. Previously published studies have shown that RUNX2 interacts specifically with hypophosphorylated pRB during initiation of cell-cycle withdrawal during terminal osteoblast differentiation [118, 121, 133]. Inactivation of pRB is very common to a small subset of tumours including osteosarcoma [134], and in particular, 50%–70% of osteosarcomas do not have functional pRB [6]. In the absence of pRB, RUNX2-pRB-induced cell-cycle exit would not be possible, and this could lead to uninhibited proliferation of osteoprogenitor cells, as well as increased genomic instability [135].

Apart from the pRB–RUNX2 connection, there is evidence indicating that normal RUNX2 function in bone is linked to the p53-MDM2 pathway [136]. The p53 pathway is perturbed in Li-Fraumeni patients, and there is increased osteosarcoma incidence in Li-Fraumeni families [8, 9]. Furthermore, bone-specific knockout of p53 is dominant over loss of pRB in the predisposition to osteosarcoma in mouse models [119, 137]. RUNX2-dependent osteoblastic differentiation is compromised when the p53-MDM2 pathway is genetically perturbed, and loss of p53 function increases the differentiation-related accumulation of RUNX2 [138]. In contrast to primary or immortalised osteoblasts, which normally have low RUNX2 levels, loss of p53 correlates with elevated RUNX2 protein levels in several growth factor-independent osteosarcoma cell lines [26, 27]. Hence, it is conceivable that loss of p53 function in osteosarcomas is permissive for or even contributes to the elevated protein levels that are observed in osteosarcoma patient samples with 6p12-6p21 gene amplifications [11–17, 22]. 

Cell cycle-dependent activity of RUNX2 is regulated by cyclin-dependent kinase- (CDK-) mediated phosphorylation [89], and the p27*^KIP1^*/CDKN1B cyclin-dependent kinase inhibitor is also required for terminal differentiation and cell-cycle exit by interaction with RUNX2. Protein levels of p27*^KIP1^* are reduced in the undifferentiated subtype of osteosarcoma [118]. Our own aCGH analysis of 15 osteosarcoma patient samples detected loss of *CDKN1B *in nine of 15 samples (our unpublished data). RUNX2 signaling in the absence of the tumour suppressors pRB and p27*^KIP1^* would, therefore, be limited in its capacity to halt proliferation and induce osteoblast maturation. Similarly, reduced expression of the p21*^CIP1^*/CDKN1A cyclin-dependent kinase inhibitor may occur as a result of elevated RUNX2 protein levels (which transcriptionally represses the p21*^CIP1^*/CDKN1A gene) [110] and the concurrent loss of p53 (which is the major transactivator of p21*^CIP1^*/CDKN1A)[139]. Reduced p21*^CIP1^* levels would prevent cell-growth arrest and DNA repair following DNA damage during chemotherapy and radiation of osteosarcomas in the clinic. 

Clearly, the prodifferentiation and tumour suppressor function of RUNX2 has potential for deregulation, in that MSCs committed to the osteoblast lineage could be stalled in their differentiation before development of the mature osteoblast phenotype. Recently, it was found that Notch1 inhibits RUNX2 directly by binding it [140] and indirectly by upregulating cyclin D1-dependent kinase CDK4, which ubiquitinates RUNX2 [141]. An association has been found between upregulated Notch signaling and lung metastatic potential in osteosarcoma cell lines [142], but no functional studies have yet linked inactivation of RUNX2 directly to osteosarcoma metastasis.

Contrary to the tumour suppressor-like behaviour of RUNX2 that has been described by previously published studies of the protein [24, 104, 143], several recent studies have identified RUNX2 as potentially having a direct role in promoting neoplasia, particularly in prostate and breast cancers. To begin with, RUNX2 is highly integrated, often through reciprocal activation pathways, with PI3K/Akt, Wnt, BMP/TGF*β*, MAPK/ERK, and Notch signaling, all of which can be activated in osteosarcomas and other tumours [144–147]. A comprehensive study by Akech et al. [148] demonstrated that overexpression of *RUNX2* in prostate cancer cells inoculated into bone led to activation of genes necessary for osteolytic disease, *PTH-related protein* (*PTHrP*) and *interleukin 8* (*IL8*). Both PTHrP and RUNX2 activate expression of receptor activator of nuclear factor-*κ*B ligand (RANKL), which stimulates osteoclast formation and subsequent bone resorption [149, 150] whereas IL8 promotes osteolysis through osteoclast formation independent of RANKL [151]. Interestingly, osteosarcomas are frequently mixed osteolytic and osteoblastic tumours [1], and RANK/RANKL is overexpressed in subsets of the tumours [152]. Akech et al. [148] also detected that prostate cancer overexpression of *RUNX2* activated genes necessary for metastasis and invasion (*MMP2, MMP9, MMP13*), angiogenesis (*VEGF, osteopontin*), and survival (*survivin*). These findings are consistent with other studies of the metastasis-promoting role of RUNX2 in prostate cancer cell lines [153–155] and metastatic patient specimens [156]. The results support similar observations of the requirement for *RUNX2* expression in metastatic breast cancer-associated osteolytic disease [154, 157, 158].

RUNX2 appears to have dual roles as a tumour suppressor (described above) and as an oncoprotein, depending on its cellular levels and context, and its regulation. In T-cell lymphomas, overexpression of *RUNX2 *and the *MYC *oncogene leads to cooperation between the encoded proteins that maintains survival and proliferation in the cancer cells [159]. In pituitary tumours, RUNX2 upregulates the anoikis suppressor galectin-3 (LGALS3) [160], which may also facilitate osteosarcoma metastasis [161]. The role of the protein in bone tumourigenesis is complicated, however, by incomplete knowledge of consequences of its deregulation in osteoblasts. High levels of RUNX2 inhibit apoptosis of osteoblasts in the presence of parathyroid hormone (PTH), which stimulates bone turnover [162]. Interaction between overexpressed RUNX2 and the protein product of proto-oncogene *FOS*, whose overexpression in mice led to development of the first osteosarcoma mouse model [163], upregulates transcription of the metastasis-associated gene *MMP13 *via transcription factor AP-1 [164] and has potential for other roles in oncogenesis [165].

## 8. Conclusions and Future Directions

The dual roles of RUNX2 must be tightly regulated during osteoblast differentiation for normal bone development. Other studies have noted the resemblance of some osteosarcomas to committed osteoprogenitor cells that have undergone cell-cycle deregulation and have been blocked in their differentiation towards osteocytes [118, 130, 166–168]. Additionally, there is a range of differentiation status among osteosarcomas [1] that is reflected in the well-described osteosarcoma cell lines [26, 118, 169–173] and has been demonstrated in the development of mouse models of the disease [119, 137]. Disruption of RUNX2 signaling by high levels of the protein in osteoblast progenitor cells (Figure 3(b)) could significantly interrupt osteoblast differentiation and cell-cycle regulation.

It is possible that *RUNX2* overexpression resulting from gain and amplification of chromosome 6p12-p21 is a causative factor in osteosarcoma pathogenesis, because it is consistently overexpressed in patient specimens [12, 128, 131, 132], because of its oncogenic potential, and because of the potential for its tumour suppressor functions to be deregulated. Its overexpression at the protein level is likely driven by its genetic amplification at the DNA level [12, 174], our unpublished data] and facilitated by disrupted degradation [27, 132]. The instability of chromosome 6p12-p21 that leads to *RUNX2 *gain and amplification has been demonstrated by many studies of patient samples, including biopsies [11–13, 15, 17], and thus it is probably an early event in osteosarcoma pathogenesis.

The complexity of osteosarcoma has continually posed a serious problem to understanding the etiology of the disease and identifying prognostic or predictive factors, or therapeutic targets. RUNX2 has potential to be predictive of response to the standard chemotherapy regimen according to studies by our lab, but further work to discover its cancer-specific function is needed. Additionally, larger cohorts of patients are necessary to definitively link RUNX2 level to treatment response in osteosarcoma tumours. In conclusion, the frequency of *RUNX2 *gain and elevated RUNX2 in osteosarcoma patient specimens as well as its documented functions lends to its possible value as a predictive factor and as a therapeutic target.
